# Decrease in vancomycin MICs and prevalence of hGISA in MRSA and MSSA isolates from a German pediatric tertiary care center

**DOI:** 10.1007/s15010-023-02036-5

**Published:** 2023-04-18

**Authors:** Katharina Haas, Melanie Meyer-Buehn, Ulrich von Both, Johannes Hübner, Tilmann Schober

**Affiliations:** 1 Division of Pediatric Infectious Diseases, Dr. Von Hauner Children’s Hospital, Ludwig-Maximilians-University Munich, Munich, Germany; 2 German Center for Infection Research (DZIF), Partner Site Munich, Munich, Germany; 3 Division of Pediatric Infectious Diseases, Montreal Children’s Hospital, McGill University, McGill University Health Centre, 1001 Décarie Blvd, Montréal, QC H4A 3J1 Canada; 4 Division of Medical Microbiology, McGill University, Montreal, Canada

**Keywords:** Vancomycin, Minimum inhibitory concentration, Heterogeneous glycopeptide-intermediate *S. aureus*, hGISA, MRSA

## Abstract

**Purpose:**

Resistance of *Staphylococcus aureus* to vancomycin includes a general increase of minimal inhibitory concentrations (MIC) within the susceptible range over time (Vancomycin MIC Creep) and the presence of a subset of the bacterial population that expresses resistance (heterogeneous glycopeptide-intermediate *S. aureus*; hGISA). Increased MICs have been associated with adverse clinical outcomes. However, the vancomycin MIC creep is not a uniform trend suggesting the importance of regional surveys.

**Methods:**

We performed a retrospective analysis at a German pediatric tertiary care hospital. Isolates from 2002 to 2017 were selected which were newly identified methicillin-resistant *S. aureus* (MRSA) or samples from invasive methicillin-susceptible *S. aureus* (MSSA) or MRSA infections. Vancomycin and oxacillin MICs as well as GISA/hGISA were measured using MIC test strips and resistance was evaluated over time.

**Results:**

A total of 540 samples were tested, 200 from the early (2002–2009) and 340 from the later period (2010–2017). All samples were vancomycin susceptible, but the MIC was higher for the earlier samples as compared to the later ones (1.11 vs 0.99; *p* < 0.001). 14% of the samples were hGISA, GISA strains were not detected. Again, vancomycin resistance decreased over time with 28 vs. 6% hGISA (*p* < 0.001). There was no significant difference between MRSA and MSSA samples with respect to vancomycin MIC and hGISA prevalence.

**Conclusion:**

This study shows a decreasing trend for both MIC values and presence of hGISA strains highlighting the importance of monitoring local susceptibilities. Vancomycin remains a first-line treatment option for suspected severe infection with Gram-positive cocci and proven infection with MRSA.

**Supplementary Information:**

The online version contains supplementary material available at 10.1007/s15010-023-02036-5.

## Introduction

*Staphylococcus aureus* (*S. aureus*) is regarded as one of the most important pathogens in all age groups [1]. Vancomycin is a first-line treatment option for several indications due to the worldwide spread of Methicillin-resistant *S. aureus* (MRSA) infections [2]. In 1996, the first MRSA strain with reduced susceptibility to vancomycin was described in Japan [3]. The Clinical and Laboratory Standards Institute (CLSI) breakpoints defines strains with a MIC of 4–8 µg/mL as intermediate and ≥ 16 µg/mL as resistant [4]. The European Committee on Antimicrobial Susceptibility Testing (EUCAST) is more stringent and considers a MIC of ≥ 4 µg/mL as resistant since increasing the dose of vancomycin is limited by toxicities [5].

Although the occurrence of truly vancomycin-resistant strains remains extremely rare, there is concern about *S. aureus* strains that show heteroresistance to vancomycin [6]. In these cases, there is a subpopulation with a MIC of > 2 µg/mL for vancomycin within an otherwise susceptible strain. In the presence of vancomycin, selection pressure favors the proliferation of intermediate-resistant clones. Persistent vancomycin exposure can lead to a uniform GISA population [7]. Importantly, this mechanism is different from fully vancomycin-resistant strains that have acquired plasmid-encoded *vanA* from *Enterococcus* species [8].

Clinically, the treatment of hGISA poses a major problem, as it is not possible to detect the resistance using standard methods. In addition, infections with hGISA are associated with poor treatment outcome [9].

Another problem in the treatment of infections with MRSA is a phenomenon known as "Vancomycin Minimal Inhibitory Concentration Creep" (Vancomycin MIC Creep) [6]. This refers to a progressively increasing tendency of MIC in *S. aureus* strains, which are in the susceptibility range according to current guidelines [10]. Elevated vancomycin MIC levels even within the susceptibility range are associated with poor treatment outcomes, including delayed drug response, prolonged hospitalization and increased mortality rates [11, 12]. In addition, higher vancomycin MICs are strongly correlated with the likelihood of becoming a hGISA strain [13].

Importantly, vancomycin MIC Creep is not a uniform trend globally [14]. It exists in some centers, whereas most centers report unchanged or even declining vancomycin MICs in *S. aureus* [15]. Pediatric studies have shown similarly conflicting results and data—especially from Europe—is scarce [10, 16].

The primary aim of this study was to evaluate the temporal trend of vancomycin MIC values and the potential presence of a vancomycin MIC creep in a central European tertiary care children’s hospital. Secondly, the strains were tested for heterogeneous and intermediate resistance to glycopeptides. Thirdly, we assessed the temporal development of methicillin resistance and its association with vancomycin MIC.

## Materials and methods

### Patient population and sampling

In this retrospective study, 540 bacterial isolates were evaluated that had been taken from patients or patient’s caregivers of a pediatric tertiary hospital from May 2002 to July 2017. Only one isolate per patient was used for evaluation within a timeframe of six months. The isolates have been frozen after initial detection of MRSA or severe *S. aureus* infections (blood stream infection, endocarditis, pneumonia, and osteomyelitis). The samples from adults were collected as part of an infection control program that screened the caregivers of children with MRSA or severe *S. aureus* infections. Criteria for storage had been prospectively defined. This study was performed in line with the principles of the Declaration of Helsinki. Ethical approval was obtained from the ethics committee of the LMU Munich (ID 21-0334).

Isolates were stored using the Cryobank system (MAST Diagnostica, Reinfeld, Germany). For recultivation, the cultures were plated on Columbia agar plates with 5% sheep blood (Becton Dickinson, Heidelberg, Germany). 13 Isolates did not grow on the agar plates, and were not used for further evaluation.

### Cultivation and resistance determination

The MIC was measured using the E test (Liofilchem, Roseto degli Abruzzi, Italy) method. Bacterial isolates were grown on Columbia agar plates and incubated overnight at 37 °C. Suspensions were prepared to a 0.5–1 McFarland turbidity standard and plated on Mueller—Hinton agar plates (Becton Dickinson, Heidelberg, Germany). After a rest period of 15 min at room temperature, MIC testing was performed following manufacturer’s guidelines. MICs were evaluated after 24 h. The isolates were tested for vancomycin and oxacillin. For the detection of heterogeneous glycopeptide-intermediate *S. aureus* (hGISA) and glycopeptide-intermediate *S. aureus* (GISA) we used Glycopeptide Resistance Detection (GRD; Liofilchem, Roseto degli Abruzzi, Italy) strips. This test has been extensively validated [17, 18]. The following well-characterized *S. aureus* strains were used as controls for internal verification according to the manufacturer’s recommendations: ATCC 29213; ATCC 700698 (Mu3; hGISA; Vancomycin MIC 3 mg/L) [19] and ATCC 700699 (Mu50; GISA; Vancomycin MIC 8 mg/L) [19]. The measured MIC values were classified using EUCAST criteria with a cutoff of 2 µg/mL for both vancomycin and oxacillin.

### Proportion of MRSA and MSSA

For methicillin resistance, antibiograms from the diagnostic routine of 15,852 patients were evaluated over a time period from 2002 to 2017. Until 2011, the microorganisms were identified using API ID test strips (bioMérieux, Marci-l’Étoile, France), Oxacillin plates (Becton Dickinson, Heidelberg, Germany) and the Mec-A test (Oxoid, Hampshire, UK). Since 2011, resistance has been determined with the Vitek system (bioMérieux, Marci-l’Étoile, France), Oxacillin plates (Becton Dickinson, Heidelberg, Germany) and the Mec-A test (ThermoFisher, Wesel, Germany).

### Data source and statistical evaluation

Data were analyzed using Excel 16.0 (Microsoft, Redmond, USA). Statistical significance between two study groups was calculated using the Mann–Whitney *U* Test (Mann, Whitney 1947). Frequencies of nominally scaled variables were evaluated using the Chi-square test (*x*^2^ test; Pearson 1900). Both tests were calculated with the Excel 16.0 program. The significance levels were set at *p* ≤ 0.05 = significant (*), *p* ≤ 0.01 = very significant (**) and *p* ≤ 0.001 = highly significant (***).

## Results

### Susceptibility of isolates to vancomycin

A total of 540 *S. aureus* isolates obtained from 531 patients underwent susceptibility testing for vancomycin and oxacillin. The age range of the patients was between 0 and 52 years. 81% of the samples came from patients under 18 years. Of these, 34% were younger than one year at the time of sampling. 200 isolates could be assigned to an early time interval from 2002 to 2009 and 340 isolates to a late time interval from 2010 to 2017. Ninety percent of the samples were newly identified MRSA isolates, 10% of the samples came from invasive *S. aureus* infections (Supplementary Fig. 1).

**Fig. 1:**
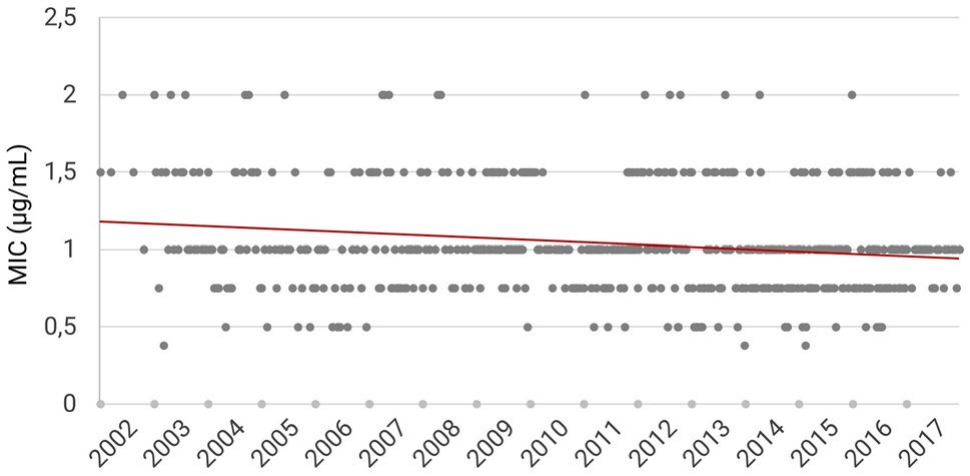
Distribution of MIC values and trend line over time indicating decreasing vancomycin MIC in *S. aureus*-isolates (*R*^2^ = 0.0279). *MIC* minimal inhibitory concentration

All MIC values for vancomycin were within the susceptible range according to EUCAST criteria. MIC_50_ was 1 µg/mL, MIC_90_ 1.5 µg/mL. During the study time, we detected a decrease of the average MIC values which accounted for 0.0144 µg/mL/year (Fig. 1).

**Fig. 2:**
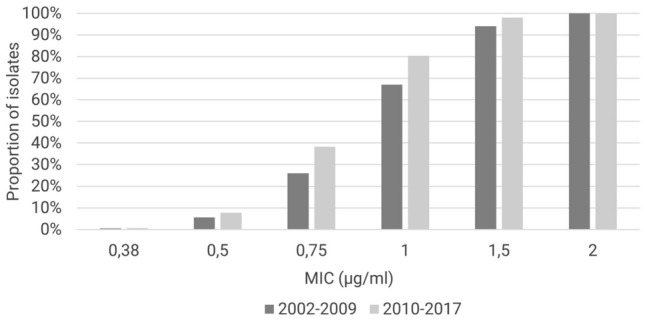
Cumulative percentage of vancomycin MIC per time period showing significantly lower MICs in *S. aureus* isolates from the recent period after 2010 (*p* < 0.001). *MIC* minimal inhibitory concentration

The comparison between early and late time interval also shows decreasing of MIC. Eighty percent of the samples selected since 2010 showed a MIC ≤ 1 µg/mL as compared to 67% before 2010. The mean value of the MIC values was 1.1 µg/mL in the early and 0.99 µg/mL in the late time interval (*p* < 0.001; U test; Fig. 2).

**Fig. 3:**
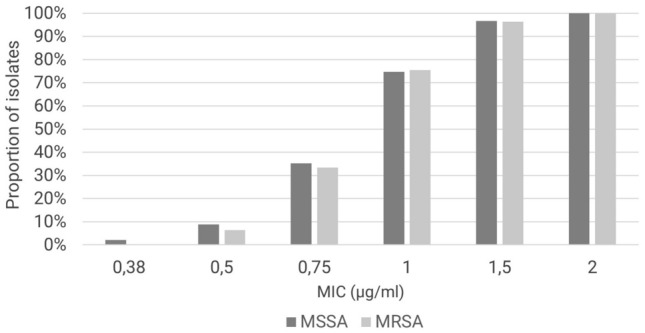
Comparison of cumulative percentages of vancomycin—MIC between MSSA and MRSA samples. *MIC* minimal inhibitory concentration

We found 83% of the samples to be resistant to methicillin and the remaining 17% were MSSA isolates. The frequency of vancomycin MIC values did not differ between MRSA and MSSA isolates; (*p* = 0.79; U test; Fig. 3). Mean and median MICs were also comparable (1 µg/mL in all groups). The temporal trend of MIC values in this study is independent of the patient age. The difference in MIC values between isolates of patients under 18 years of age and those of patients 18 years and older was not statistically significant (data not shown).

### Heterogeneous glycopeptide-intermediate *S. aureus*

**Fig. 4:**
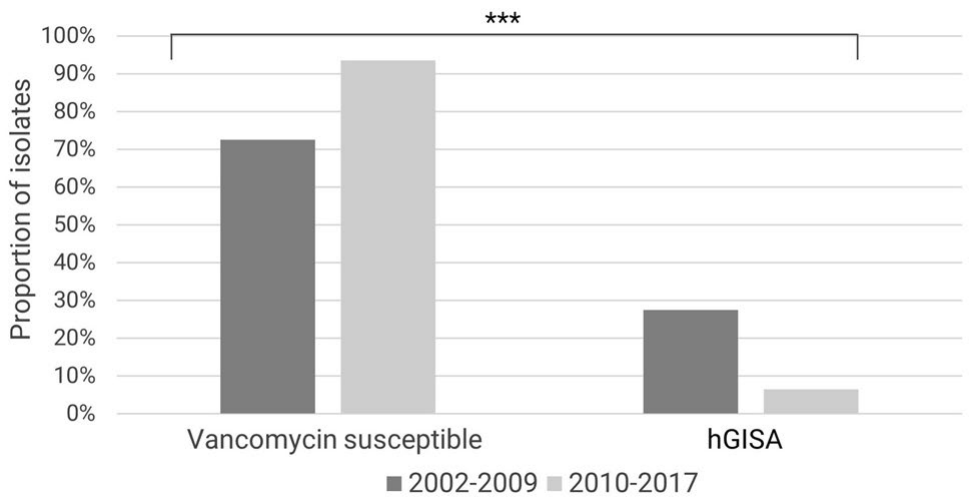
Percentage of susceptible and hGISA strains per time period demonstrating decreasing heterogeneous intermediate glycopeptide resistance

There were no samples with intermediate glycopeptide resistance. As much as 14% of all samples showed heterogeneous resistance to glycopeptides. This rate declined during the investigated period. However, there were substantial fluctuations with a share of hGISA between 0 and 40% per year. A comparison between early and late time intervals also shows a decreasing trend of hGISA strains from 28 to 6% (*p* < 0.001; *x*^2^; Fig. 4). There were only minor differences in intermediate resistance of MRSA and MSSA isolates: the proportion of hGISA was 15% for MRSA isolates and 11% for MSSA isolates (*p* = 0.33; *x*^2^).

There was a strong correlation between vancomycin MIC values and the hGISA phenotype. High vancomycin MIC values were positively correlated with heterogeneous resistance to glycopeptides. Although the mean MIC of susceptible strains was 1 µg/mL, the mean value of hGISA was 1.2 µg/mL (*p* < 0.001; *U* test).

### Susceptibility of *S. aureus* to methicillin

**Fig. 5:**
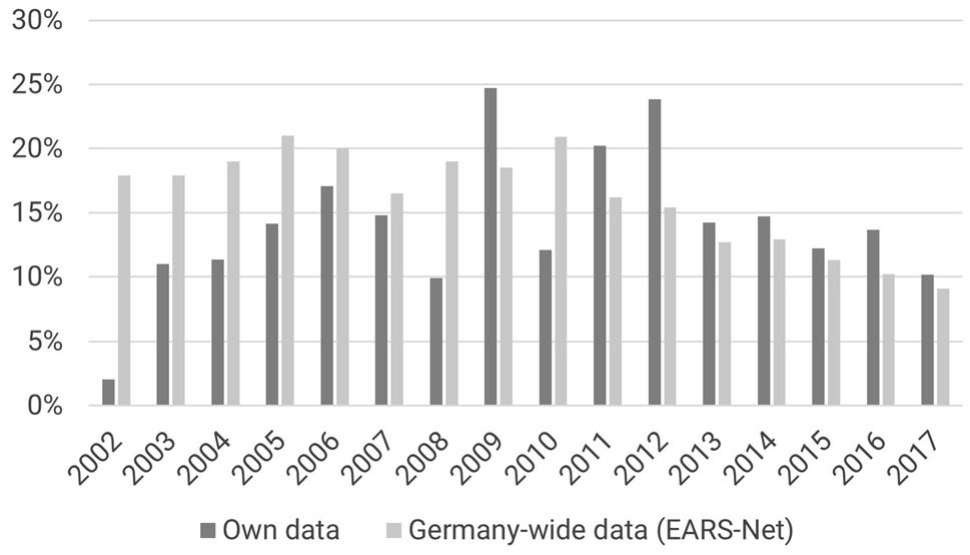
Proportion of MRSA strains in the present study as compared to data from the EARS-Net (Germany only [20–22])

Antibiograms of 15,852 patients from the same time period (2002–2017) were evaluated to independently assess methicillin susceptibility. In total, 14% of the *S. aureus* samples were methicillin resistant. The percentage increased until 2009 and has deceased since (Fig. 5). These were in line with the national German data. The European Antimicrobial Resistance Surveillance Network (EARS-Net) reports a mean methicillin resistance rate of 16.2% from Germany for *S. aureus* isolates from blood cultures over the same period from 2002 to 2017 [20–22]. Similar to our study, the rate increased until 2010 and has decreased since (Fig. 5).

## Discussion

We were able to show a decreasing trend for both the *S. aureus* vancomycin MIC and the presence of hGISA strains over a 15-year period in a German pediatric tertiary care center. Accordingly, we did not see a vancomycin MIC creep, but rather the opposite.

Similar to our data, a Vancomycin creep could not be confirmed in the largest study to date by SENTRY Antimicrobial Surveillance Program with isolates from 45 countries from 1997 to 2016 and in a recent metaanalysis [14, 23]. However, there are pronounced regional differences and data quality is heterogeneous. Estimates for hGISA prevalence are ranging from 1.3 to 27% [24–26]. Our hGISA rate of 14% is within this range. The marked differences are attributed to varying detection methods and origin of isolates, as well as prior exposure to vancomycin. Insufficient standardization of the respective test methods is a challenge when comparing of hGISA prevalence at different centers. Three different methods have been validated and are routinely used, namely, population analysis profile area under the curve ration (PAP-AUC), the macroversion E test, and the GRD E test which we used—none of them are commonly available in routine laboratories [17, 18, 27]. Long-term studies using the same methodology like the presented study are still important for study of trends over several years. Meta-analyses of hGISA prevalence showed a decline in most studies since 2010, albeit with regional differences [25, 26]. Namely, the world’s most populous countries India and China continue to have high rates [25, 26].

There is little data regarding MRSA prevalence in pediatric populations in Germany highlighting the importance of the presented study. The decreased rate of methicillin-resistant MRSA since 2009 in our dataset and in the German EARS-Net is part of a general European trend. The European average also shows a continuously decreasing trend from 23.2% in 2009 to 16.9% in 2017, although this trend is not uniform, and there are also countries with increasing MRSA rates [20, 22, 28]. Globally, there are substantial differences. Importantly, MRSA prevalence does not seem to be improving in countries that also have high Vancomycin MICs and hGISA rates, such as India and China [29, 30].

The mechanism of hGISA is different from that of MRSA, with cell wall thickening and reduced vancomycin access to its active site being the main factors as compared to acquisition of a non-native gene encoding a penicillin-binding protein [27]. However, hGISA is not independent of MRSA. In fact, hGISA development is considered to be an indirect consequence of resistance to beta-lactam antibiotics [6, 25, 27, 31]. Most but not all hGISA strains are also methicillin resistant [25, 31]. Suspected or confirmed MRSA infections are the most important indication for vancomycin use. In this way, higher MRSA frequencies are associated with higher Vancomycin use and subsequent evolutionary pressure favoring higher Vancomycin MICs and hGISA development [6, 14, 25, 31]. This sequence has not only been shown in epidemiological studies, but also intra-individually [32, 33]. Importantly, typing of hGISA strains have identified clonal lineages [27, 31]. These have developed independently and different clones have been associated with local and regional outbreaks, such as LIM-2 in Western Europe, USA-100 in North America, or Mu50 in Japan [27, 31, 34, 35]. Fortunately, hGISA clones seem to have lower growth rate [7]. Accordingly, higher awareness and appropriate measures against MRSA, such as routine MRSA screenings and improved infection control measures in health care institutions, have likely contributed to the observed decline in hGISA prevalence [25]. It is encouraging and should be emphasized in antibiotic stewardship programs that targeted interventions can have broader beneficial effects on antibiotic resistance. Accordingly, the positive trend seen in Germany could be replicated in large countries with higher hGISA rates such as China and India. However, a detailed evaluation of these measures is difficult and it is impossible to prove causality.

Our study has several limitations. First, it is a retrospective single-center study. Second, antibiotic exposure and clinical treatment outcomes were not correlated with antibiotic susceptibility. Third, the hGISA and GISA phenotypes are unstable and might thus drop to a lower resistance level by eliminating the selection pressure. Accordingly, storage of isolates potentially affects susceptibility testing results [16, 27, 36]. However, we do not expect this to bias our analyses since all samples were treated identically. Forth, MIC values were only measured by one method—the E test—which can show subjective measurement inaccuracies. Fifth, strains were not genotyped. Accordingly, the impact of specific clones could not be assessed although there was no indication of any outbreak or significant nosocomial transmission. Sixth, the data on the susceptibility of *S. aureus* to methicillin over time is derived from a different patient cohort than the Vancomycin MIC and hGISA prevalence. Nevertheless, the two cohorts are from the same institution and same time period and are representative for the reported trends over time.

In summary, vancomycin remains suitable as first-line treatment for an expected severe infection with Gram-positive cocci and proven MRSA infections. However, a continuous analysis of regional conditions is essential as they may differ from global trends. The regular monitoring of vancomycin MIC is advisable—additional screens for hGISA and GISA do not seem routinely required as they are closely correlated with vancomycin MIC.

## Supplementary Information

Below is the link to the electronic supplementary material.

Supplementary file1 (DOCX 16 KB)

## Data Availability

The data that support the findings of this study are available on upon reasonable request from the corresponding author with a research protocol and ethical approval.
