# Supplementary material for: Decrease in vancomycin MICs and prevalence of hGISA in MRSA and MSSA isolates from a German pediatric tertiary care center
Source: Infection. 2023 Apr 18;51(3):583–8. doi: 10.1007/s15010-023-02036-5 (PMC10205833; doi:10.1007/s15010-023-02036-5)
Supplement: Supplementary file 1 — Supplementary file1 (DOCX 16 KB) [file 15010_2023_2036_MOESM1_ESM.docx]

# Supplementary Information: Decrease in vancomycin MICs and prevalence of **hGISA** in MRSA and MSSA isolates from a German pediatric tertiary care center

**Supplementary Figure 1:** Collection sites of isolates and classification into invasive and noninvasive samples:

|  | Airways | Eye | Blood stream | Gastro- intes-tinal | Skin /  Soft tissue | Bone / joint | Uro-genital | CNS | Wound | Central  line |
| --- | --- | --- | --- | --- | --- | --- | --- | --- | --- | --- |
| Frequency: | 334 | 8 | 39 | 46 | 44 | 3 | 4 | 4 | 54 | 4 |
| (%) | 62 | 1% | 7% | 9% | 8% | 1% | 1% | 1% | 10% | 1% |
| Classification |  |  |  |  |  |  |  |  |  |  |
| Invasive: | 1 | 0 | 39 | 1 | 0 | 3 | 1 | 4 | 0 | 4 |
| (%) | 2% | 0% | 74% | 2% | 0% | 6% | 2% | 8% | 0% | 8% |
| Non-invasive: | 333 | 8 | 0 | 45 | 44 | 0 | 3 | 0 | 54 | 0 |
| (%) | 68% | 2% | 0% | 9% | 9% | 0% | 1% | 0% | 11% | 0% |
